# Spatially selective photoconductive stimulation of live neurons

**DOI:** 10.3389/fncel.2014.00142

**Published:** 2014-05-21

**Authors:** Jacob Campbell, Dipika Singh, Geoffrey Hollett, Shashank M. Dravid, Michael J. Sailor, Jyothi Arikkath

**Affiliations:** ^1^Developmental Neuroscience, Munroe-Meyer Institute, University of Nebraska Medical CenterOmaha, NE, USA; ^2^Materials Science and Engineering Program, University of CaliforniaSan Diego, La Jolla, CA, USA; ^3^Department of Pharmacology, Creighton UniversityOmaha, NE, USA; ^4^Department of Chemistry and Biochemistry, University of CaliforniaSan Diego, La Jolla, CA, USA

**Keywords:** photoconductive stimulation, primary neurons, non-invasive neuronal stimulation, spatially selective stimulation, couple with live imaging

## Abstract

Synaptic activity is intimately linked to neuronal structure and function. Stimulation of live cultured primary neurons, coupled with fluorescent indicator imaging, is a powerful technique to assess the impact of synaptic activity on neuronal protein trafficking and function. Current technology for neuronal stimulation in culture include chemical techniques or microelectrode or optogenetic based techniques. While technically powerful, chemical stimulation has limited spatial resolution and microelectrode and optogenetic techniques require specialized equipment and expertise. We report an optimized and improved technique for laser based photoconductive stimulation of live neurons using an inverted confocal microscope that overcomes these limitations. The advantages of this approach include its non-invasive nature and adaptability to temporal and spatial manipulation. We demonstrate that the technique can be manipulated to achieve spatially selective stimulation of live neurons. Coupled with live imaging of fluorescent indicators, this simple and efficient technique should allow for significant advances in neuronal cell biology.

## Introduction

Advances in light microscopic techniques and availability of a variety of fluorescent indicators for examining protein trafficking and cellular function in live neurons has led to significant advances in our understanding of the cellular changes that underlie neuronal structure and function (Granger et al., 2013; Karpova et al., 2013). Several types of neurons, including cortical, hippocampal (Li et al., 2010; Sylwestrak and Ghosh, 2012) and cerebellar granule neurons (Kim et al., 2009), are highly amenable to being cultured in a primary neuronal cell culture system (Beaudoin et al., 2012), thus allowing cell biological studies in a neuron type specific manner. These culture systems are well established and have been used by several groups to make fundamental advances in neuronal cell biological studies (Aoto et al., 2013; Siddiqui et al., 2013).

Neurons are specialized electrically excitable cells in which synaptic activity is a key regulator of neuronal structure and function (Ebert and Greenberg, 2013). Synaptic activity is a critical mediator of inter-neuronal communication and can regulate a variety of intracellular signaling cascades, including those related to short-term and long-term memory (Bloodgood et al., 2013). Thus, the combination of live cell imaging techniques coupled with the ability to stimulate live neurons has great advantages for neuronal cell biological studies (Okada et al., 2009).

Current approaches to manipulate synaptic activity in cultured primary neurons include chemical techniques [e.g., KCl (Aizawa et al., 2004; Grubb and Burrone, 2010), glutamate receptor agonists and antagonists (Anggono et al., 2011)]. However, these have the disadvantage of lack of user control on spatial resolution without specialized equipment. Other techniques that allow for neuronal stimulation include current injection using microelectrodes and growing cells on specially patterned microelectrode arrays (Kutzing et al., 2011; Bakkum et al., 2013). Optogenetic techniques have also been used for such studies, however, they require introduction of exogenous DNA into the neurons and Light Emitting Diodes or other light sources for stimulation (Lin et al., 2013; Watanabe et al., 2013). Thus, these techniques, while powerful, have the disadvantage of being rather specialized, requiring dedicated equipment and expertise or being invasive. Moreover, they are not easily amenable to quick and easy alterations in spatial resolution that can allow the stimulation of a single or groups of neurons within the neuronal network during the same experiment in a non-invasive manner.

Photoconductive stimulation is a technique which takes advantage of the ability of light to alter the conductivity of silicon (Goda and Colicos, 2006; Hung and Colicos, 2008; Gutierrez et al., 2009; Pavlov et al., 2010). By culturing neurons on a silicon based substrate and taking advantage of photoconductive stimulation, neurons can be stimulated in a non-invasive manner. We have significantly advanced a technique for photoconductive stimulation (Colicos et al., 2001) of live cultured neurons. These advances include (a) a chamber design for an inverted microscope, (b) use of a laser induced photoconduction using a confocal microscope, (c) characterization of photoconductive stimulation of neurons on three silicon based substrates (d) manipulations that allow spatial selectivity of stimulation.

This allows for use of the photoconductive stimulation technique in an inverted confocal microscope using laser based stimulation and provides access to a technique with variable spatial resolution and relative ease of operation. We demonstrate stimulation on simple polished silicon and on surface-modified porous oxidized and porous carbonized silicon with the spatial resolution of the stimulation being dependent on the type of silicon substrate and the area of illumination.

## Materials and methods

### Cell culture

Rat cortical neurons were isolated from E18 rat embryos as previously described. These neurons were frozen using a proprietary freezing medium. Frozen neurons were thawed at 37°C and their cell viability was estimated by light microscopy. This was done by visual examination or by taking advantage of trypan blue staining. Neurons were plated at a density of 150,000 neurons on a 200 mm^2^ silicon chip or at 50,000 in a 35 mm glass bottom dish (Mattek). Both substrates were coated overnight with poly-lysine as previously described. Coated chips or dishes were washed 3 times with autoclaved water, and pre-warmed plating media was added. Thawed cells were added at the required density and incubated for 3–6 h at 37°C. Subsequently, the plating media was replaced with maintenance media. AraC (1-b-d-arabinofuranosylcytosine) was added to cultures in about 48 h after plating to limit growth of mitotic cells. Neurons were maintained in maintenance media until used on DIV 12-15. The compositions of the media are as previously described (Beaudoin et al., 2012).

### Silicon chips

Single side polished, P type, boron doped, 10–20 ohm^*^cm, (100) orientation, 500 μm thick silicon wafers (University Wafer) were cut into 14 mm square chips (1.96 cm^2^) by scoring with a diamond tipped pencil and snapping on a hard edge. Cut chips were kept in 100% ethanol until needed, then rinsed several times with fresh 100% ethanol, put into sterile 12 well plates, and allowed to air dry before coating with poly-lysine and plating cells as described above.

### Generation of silicon wafers with surface texturing

To prepare porous silicon substrates, *p*-type silicon wafers were mounted into an aluminum-backed Teflon etch cell with an exposed area of 8 cm^2^. Wafers were cleaned with ethanol before performing a sacrificial etch at 600 mA for 15 s in a 3:1 HF:Ethanol solution. The sacrificial porous layer was then removed via 2M KOH until bubbles stopped forming. A second etch was then performed at 400 mA in 3:1 HF:Ethanol for 300 s. Samples were then washed three times with ethanol and once with hexane before drying under a stream of nitrogen. Porosity and thickness of etched silicon was found to be 42% and 1900 nm respectively via the Spectroscopic Liquid Infiltration Method using a Bruggeman model [1]. Lightly oxidized samples were prepared by oxidation in air at a final temperature of 300°C for 20 min after a ramp rate of 10°C/min. Carbonized samples were prepared by briefly oxidizing freshly etched samples with ozone and then spin-coated with a carbon precursor mixture of furfuryl alcohol, oxalic acid and ethanol. The sample was then placed under flowing argon and held at 200°C for 2 h. The temperature was then increased at a rate of 5°C/min until a final temperature of 800°C was reached and then held for 1 h.

### Imaging experiments

Imaging experiments were carried out on a LSM700 (Zeiss) confocal microscope, using a Plan-Apochromat 40x/1.3 Oil DIC UVVIS-IR oil immersion objective for glass bottom dishes and a EC Plan-neofluar 20x/0.50 WD = 2 M27 objective for silicon chips. Imaging was done at 37°C, 5% CO_2_ and 95% air in a H301 top stage incubator (Harvard Apparatus). Cell cultures were immersed in external bath solution (EBS) (3.0 mMCaCl_2_, 2.0 mM MgCl_2_, 135 mM NaCl, 5.0 mM KCl, 10 mM Glucose, 5.0 mM HEPES, pH 7.3) for all experiments. Not all areas of chips made from porous silicon were suitable for experiments. This is due to the non-uniform surface of the samples we used. For porous oxidized silicon, some areas fluoresced green under 488 nm excitation, while others did not. Areas that did fluoresce were unsuitable for photoconductive stimulation, probably due to the thicker insulating oxide layer on these areas. Stimulation was effective in non-fluorescent areas. For porous carbonized silicon, some areas appeared black to the naked eye. No cells were found to grow in these areas, so they were unsuitable for stimulation. Stimulation was successful in every area with cell growth, though carbonized chips required about 10x the voltage of other chips.

### Field stimulation

Field stimulation was carried out in 35 mm glass bottom dishes, using a custom 3d printed insert (Shapeways). This insert held two parallel platinum wires 5 mm apart on the bottom of the dish. Stimulation was delivered by a Grass S48 at 10V and variable frequency.

### Photoconductive stimulation

Photoconductive stimulation was carried out with a custom 3d printed chamber (Shapeways, ZoomRP). Stimulation was targeted using the imaging lasers of an LSM 700 confocal microscope. For imaging green fluorescence, the 488nm laser was used at an appropriate power level. In order to induce photoconductivity, the 555 nm laser was used simultaneously at 70% power. This laser intensity was found empirically, by observing the formation of bubbles from water hydrolysis on the chip surface at the point of stimulation as 8 V pulses were applied and laser power increased. The power level was found at a voltage slightly above that of stimulation, in order to avoid hydrolysis during experiments. Stimulation was delivered as 2 ms long, 4–7 V square pulses at adjustable frequency from a Grass S48 stimulator. Voltage was set for each batch of chips by observing the needed level for Fluo-4 firing.

### Frequency calcium imaging

Neuronal activation was visualized by calcium imaging with Fluo-4 AM (Molecular Probes). Dye was prepared at 2–5 mM with 0.02% Pluronic F-127 (Molecular Probes) in EBS. Cells were loaded for 20–30 min at 37°C, washed once with EBS, then left in fresh EBS for 30 min at 37°C. Live imaging was performed on the LSM 700 condfocal microscope as described above. A ~30 ms frame time was achieved by zooming until the target cell body filled most of the field of view, then choosing a 40 × 40 pixel resolution with a line skip of 2, such that the true resolution was 40 × 20. The laser was set to scan both directions. Intensity traces were created by averaging every pixel in the frame for each time point. These were normalized for initial intensity and maximum intensity by subtracting the first frame value from each time point, then dividing each by the first frame value and the max frame value. $\left(\frac{F-F_{0}}{F_{0}*F_{max}}\;\right)$. Traces for each different stimulation frequency (1, 5, 10 Hz) were resampled to 100 Hz and were lined up in time using a MATLAB script and manually selecting the first spike. Lined up traces of each frequency were averaged together. The experiment was repeated using cells from independent cultures for each frequency.

### Spatial resolution calcium imaging

Cells were loaded with Fluo-4 as above. Spot scanning was used to target the stimulation. A before image was taken from the “spot select” dialogue just before stimulation, then the targeted cell was spot scanned for 5 s with simultaneous 10 Hz stimulation. An after image was taken just after stimulation ended. Pairs of images were converted to two layer stacks using ImageJ. Each stack was run through a MATLAB script which averaged intensity changes to create heat maps. Another script found the average fluorescence level of each cell in the field, and calculated the change in fluorescence from the before to after image. Cells that showed a 15% or greater increase were counted as activated. The experiment was repeated using cells from independent cultures.

### Immunohistochemistry

Neuron cultures on silicon chips were fixed and stained on DIV 7 or 14. Chips were fixed in 4% paraformaldehyde, 4% sucrose for 15 min at room temperature, washed with PBS, incubated with 0.1% Triton X-100 in PBS for 10–20 min, and blocked for 1 h in 5% BSA. Primary antibodies were applied overnight at 4C at the following dilutions in 1% BSA: rabbit MAP2 1:1500 (Cell Signaling #4542), mouse Tau1 1:1000, guinea pig VGLUT 1:4000 (Millipore #AB5905), mouse GAD6 1:100 (Developmental Studies Hybridoma Bank).

### Transfection

All transfections were carried out with Lipofectamine 2000 (Life technologies) as previously described (Beaudoin et al., 2012).

### Whole-cell electrophysiology

Whole-cell recordings were obtained from cultured neurons at room temperature (22–25°C). An external solution containing (in millimolars) 150 NaCl, 3 KCl, 10 HEPES, 6 mannitol, 1.5 MgCl_2_ and 2.5 CaCl_2_ (pH 7.4) was used for the recordings. Whole-cell patch recordings were obtained in voltage-clamp or current-clamp configuration with an Axopatch 200B (Molecular Devices, CA) and a pipette resistance of 5–8 mOhm. The internal solution for voltage-clamp recording consisted of (in mM) 110 cesium gluconate, 30 CsCl_2_, 5 HEPES, 4 NaCl, 0.5 CaCl_2_, 2 MgCl_2_, 5 BAPTA, 2 Na_2_ATP, 0.3 Na_2_GTP and 20 QX314 (pH 7.35). Voltage-clamp recordings were performed in the presence of 100 μM picrotoxin at a holding potential of −70 mV (not corrected for junction potential). The internal solution for current-clamp recording consisted of (in mM) 150 KMeSO_4_, 10 KCl, 0.1 EGTA, 10 HEPES, 0.3 Na_2_GTP and 0.2 Na_2_ATP (pH 7.3). Whole-cell recordings with a pipette access resistance less than 20 mOhm and that changed less than 20% during the duration of recording were included. Signal was filtered at 2 kHz and digitized at 10 kHz using an Axon Digidata 1440A analog-to-digital board (Molecular Devices, CA).

### Additional methods for figures

#### Figure 3

**(A–C)** were created using the 3D modeling program SketchUp 2013. A similar model from this program was used to create the physical 3D printed chamber. The model displayed in this figure has had some dimensions exaggerated to make the design easier to see. **(D)** is a photograph of one of the chambers printed and used for experiments.

#### Figure 4

Time series LSM image stacks were imported into MATLAB. Traces were created by averaging pixel intensities across each entire image for each time point. Most images had a ~34 ms frame time, but traces were linearly upsampled to 100 Hz to set a completely uniform sampling rate. Due to imaging and electrical stimulation being turned on by hand, the onset of stimulation differed in time in different image stacks. A MATLAB script displayed each trace in turn to an operator, that operator identified the position of the first spike and the script truncated each trace to begin 0.5 s before that first spike, i.e., the onset of stimulation. Traces were then normalized using the formula $\left(\frac{F-F_{0}}{F_{0}*F_{max}}\;\right)$, thus making the lowest point of each trace equal 0 and the highest point equal 100. This eliminates differences in starting intensities of each cell and differences in the amount of intensity rise from stimulation. Frequency information is in relative changes in fluorescence, so we can discard absolute intensity. For each independent culture, all the traces for each frequency were averaged together. This resulted in each independent culture having one trace for each frequency. These were then averaged together to create the traces displayed in panels **(A–C)**, **(E–G)**, **(I–K)**, and **(M–O)**. The error bars represent the standard error from the independent cultures. The images in panels **(D)**, **(H)**, **(L)**, and **(P)** are representative of 1 cell at 1Hz stimulation. These have had contrast enhanced by setting the “contrast” option in ZEN 2010 (Zeiss) to best fit and applying the LUT “rainbow 2.” The first image of each series is just before the first simulation pulse, the second is just after. Similarly for the 3rd and 4th images and the second stimulation pulse, and the 5th and 6th image and the third stimulation pulse. These images have had their resolution increased for display.

#### Figure 5

Before images were taken using the “spot select” option in ZEN 2010. The sample was then spot imaged at that point concurrently with 5 s of 10 Hz stimulation. The after image was acquired by a frame scan just after stimulation. In one experiment, whole field stimulation was achieved by stimulation during the taking of an after image. Sample before and after images are presented for each silicon type, in grayscale and the LUT “rainbow 2” (ZEN 2011). Both images were at a 512 × 512 pixel resolution, however when exported from the Zen software, the before image was RGB while the after image was 8-bit grayscale. We used ImageJ to convert the before image to also be 8-bit grayscale, then combined each pair of images into a stack, preserving scale information from the original file. A MATLAB script loaded each stack, ran a median filter on it, and stretched each histogram so that 0.1% of the after stimulation image's pixel would clip. Next the before stimulation image was subtracted from the after stimulation, and all such difference images were averaged together. This created a heat map for spot stimulation on each type of silicon and for whole field stimulation on untreated silicon. The MATLAB colormap “jet” was applied to the heatmaps over the range of 0 to the largest value found in any heatmap.

## Results

### A silicon based substrate is a permissive substrate for primary neurons

**Figure 1:**
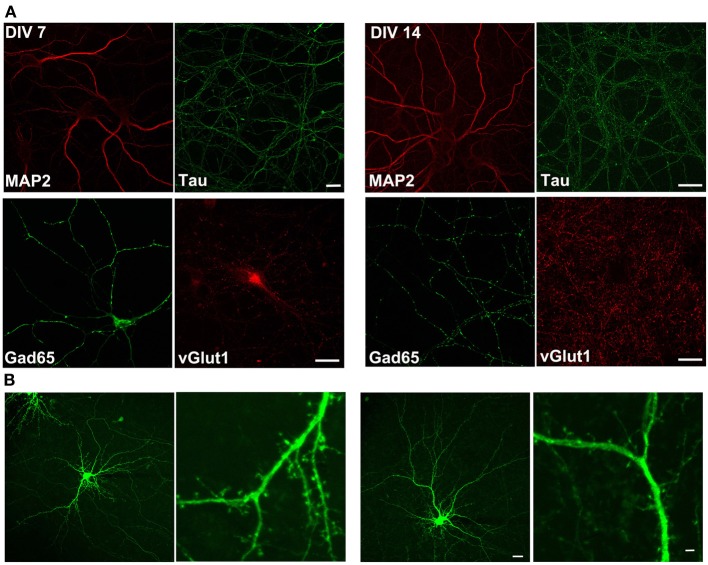
**Neurons cultured on silicon chip retain morphology and express neuronal and synaptic markers. (A)** Confocal images of cortical neurons grown on polished silicon chip and immunostained with dendritic (Map2), axonal (Tau) and synaptic (Gad65 and vGlut1) markers at DIV 7 and 14. **(B)** Confocal images of cortical neurons grown on the polished silicon chip and transfected with GFP. Note that neuronal morphology is well preserved and normal morphology of spines is observed. Scale bar 20 and 2 um.

**Figure 2:**
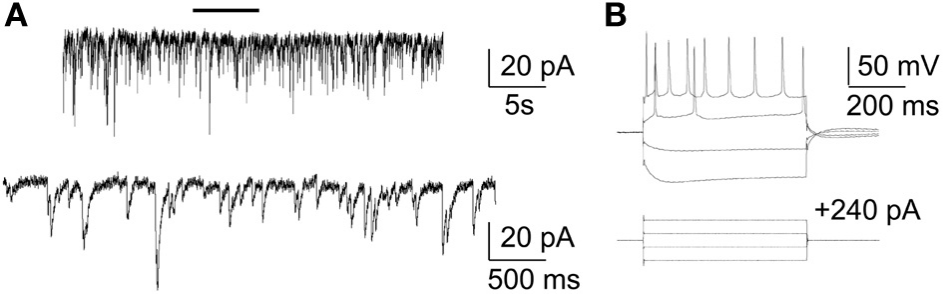
**Neurons cultured on silicon chip exhibit typical electrophysiological properties. (A)** Representative whole-cell voltage-clamp recording showing spontaneous EPSCs. Recording was performed at holding potential of −70 mV. Data were filtered at 2 kHz (1 kHz for presentation) and digitized at 20 kHz. Similar sEPSCs were observed in 3–4 neurons each from 3 independent cultures (*n* = 11). **(B)** Whole-cell current-clamp recording. The resting membrane potential of this neuron was −65 mV (not corrected for junction potential). Action potential in response to current injection. Similar results were observed in 3–4 neurons from 4 cultures (*n* = 12).

We first assessed the ability of rat primary cortical neurons to be cultured on a silicon-based substrate by immunostaining and electrophysiological techniques. The silicon based substrate, polished silicon, was coated with poly-lysine and rat primary cortical neurons were plated as previously described (Arikkath et al., 2009; Beaudoin et al., 2012). Neurons were fixed and immuostained with antibodies to neuronal and synaptic markers (Figure 1A) and transfected with EGFP to assess neuronal morphology and subjected to analysis by confocal microscopy. Neurons had normal expected morphology, expressed the dendritic marker, Map2, and axonal marker, Tau, and formed synapses as indicated by staining with excitatory and inhibitory synaptic markers, Gad65 and vGlut1. Spines also appeared to have the expected morphology (Figure 1B). In addition, we also performed electrophysiological recordings from neurons cultured on the polished silicon substrate to test the normal occurrence of spontaneous EPSCs and action potentials to assess neuronal health on this substrate (Figure 2). Neurons cultured on the silicon chip were morphologically and electrophysiologically indistinguishable from primary neurons cultured on standard glass coverslips. Thus, the silicon based substrate is a highly permissive substrate for primary rat cortical neurons.

### Design of a chamber for photoconductive stimulation on an inverted microscope

**Figure 3:**
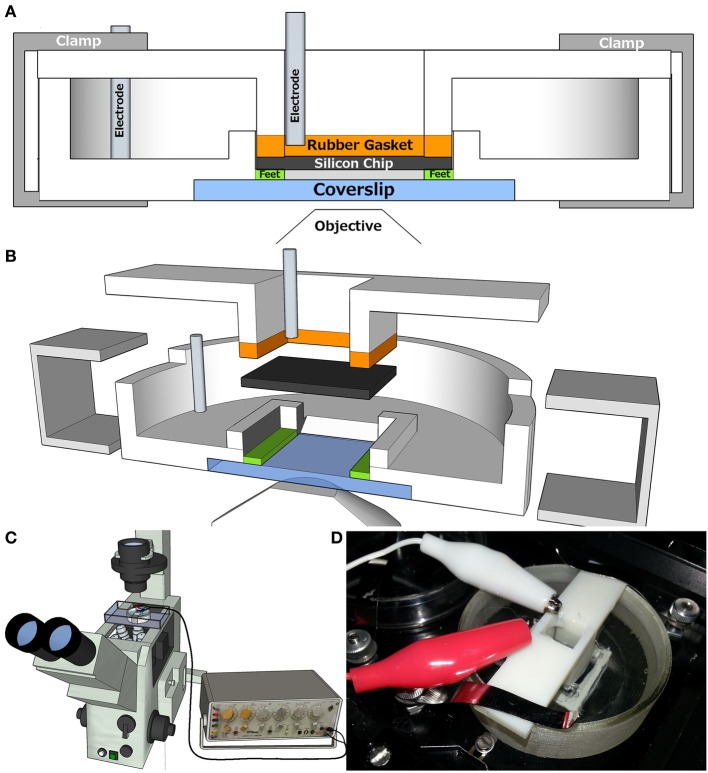
**Design and Set up of Photoconductive Stimulation Chamber for inverted microscope. (A)** Chamber is comprised of a base, silicon chip, insert, and clamps. The bottom of base chamber is a glass coverslip. Feet on coverslip keep silicon chip elevated, allowing fluid access to neurons cultured on chip. The chip is pressed onto feet by rubber gasket on insert, which is held down by clamps. This forms two chambers, in base and insert, electrically isolated by the silicon chip. Each chamber has a platinum electrode. **(B)** 3D blown-up cut away of chamber. **(C)** Chamber setup on scope. Electrodes are connected to a Grass S48 stimulator. **(D)** Photograph of chamber during experiment.

We then designed a chamber that would allow photoconductive stimulation of neurons cultured on the silicon based substrate using an inverted confocal microscope (Figures 3A–D). The inverted configuration is the configuration of choice in labs that image live neurons by light microscopy. Photoconductive stimulation uses light to lower the electrical resistance of a section of the substrate, allowing a voltage pulse applied to the back plane of the substrate to selectively shunt through the illuminated region and stimulate the cells in the near vicinity of the shunt. Unlike conventional glass and plastic substrates, silicon is opaque, which presents a challenge to inverted imaging where cells are usually imaged through their substrates. To solve this issue, our chamber holds the silicon substrate with its adherent cells suspended a small distance above a glass coverslip bottom. Thus these cells can be imaged from below by an objective with a working distance of ~400 μm. The technique can be applied either by pulsing the voltage at a constant light flux or by pulsing the laser while holding the (sub-threshold) voltage constant. The cell side and back side of the silicon chip are exposed to isolated baths and voltage pulses are delivered from an external stimulator to electrodes in these baths.

### Frequency dependent responses of neurons to photoconductive stimulation

**Figure 4:**
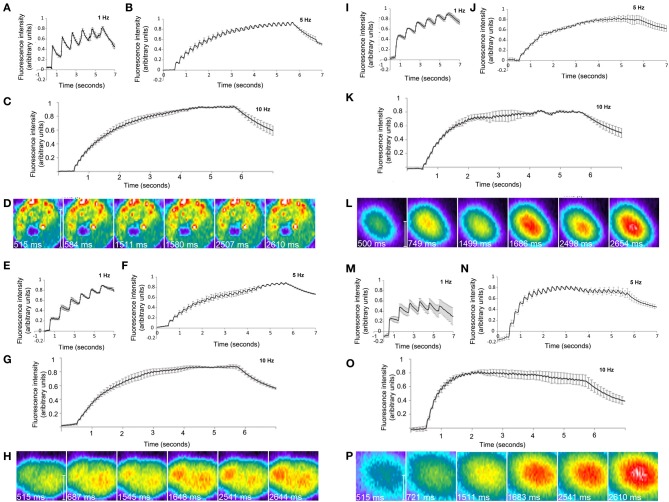
**Calcium imaging frequency response of cultured rat cortical neurons with field stimulation or photostimulation. (A)** Fluo-4 calcium imaging intensity of cultured cortical neurons in response to 5 s of 1 Hz **(B)** 5 Hz and **(C)** 10 Hz field stimulation. (*n* = 50 neurons/1 Hz, 56 neurons/5 Hz, 47 neurons/10 Hz) **(D)** Rainbow colormap images of fluo-4 based calcium response from one cell undergoing 1 Hz field stimulation. Fluo-4 calcium imaging intensity response of neurons to 5 s of 1 Hz 5 Hz and 10 Hz photoconductive stimulation on **(E–G)** polished silicon (*n* = 37 cells/1 Hz, 48 cells/5 Hz, 51 neurons/ 10 Hz), **(I–K)** porous oxidized silicon (*n* = 40 cells/1 Hz, 34 cells/5 Hz, 24 neurons/ 10 Hz) or **(M–O)** porous carbonized silicon (*n* = 37 /1 Hz, 25 cells/5 Hz,20 neurons/10 Hz). Rainbow colormap images of fluo-4 based calcium response from cell undergoing 1Hz photoconductive stimulation on **(H)** silicon chip **(L)** porous oxidized silicon or **(P)** porous carbonized silicon. Each trace in **(A–C)**, **(E–G)**, **(I–K)** and **(M–O)** represents the average of two to three independent cultures. Before averaging, the response from each cell was normalized using the formula Δ*F*/(*F*_0_*F_*max*_). Error bars show standard error. Scale bar-10 μm.

We first examined the frequency dependent responses of neurons cultured on conventional glass coverslips in a glass bottom chamber to electrical field stimulation by means of external electrodes. Neurons were loaded with the calcium indicator dye, Fluo-4 (Taylor et al., 2010) and stimulated at 1, 5, or 10 Hz (Figures 4A–D, Supplementary video 1) and changes in fluorescence intensity of individual cell bodies were measured. As expected, the response of the cells as indicated by an increase in fluorescence intensity was tightly correlated with the frequency of stimulation.

To examine the ability of neurons to be stimulated using photoconductive stimulation, we chose the approach of pulsing the applied voltage while maintaining a constant light flux. Using a similar Fluo-4 dye loading approach, we examined the ability of neurons cultured on the polished silicon substrate to elicit cellular responses in a frequency dependent manner in response to a combination of constant laser light and electrical stimulation of the silicon substrate at 1, 5, and 10 Hz (Figures 4E–H, supplementary video 2) in comparison to the similar responses elicited by field stimulation. We similarly characterized neuronal responses in neurons cultured on the silicon wafers that contained a surface texturing of mesoporous silicon oxide (Figures 4I–L, supplementary video 3) and mesoporous carbonized silicon (Figures 4M–P, supplementary video 4). Stimulation was targeted by using the digital zoom factor to restrict laser scanning to a single cell body. Photoconduction was induced by using a second laser at high power concurrently with the imaging laser. Neurons responded to stimulation with oscillatory increases in Fluo-4 fluorescence at the stimulation frequency. These response patterns were similar between all three silicon substrates and similar to those observed in neurons that were stimulated using field stimulation. Thus, the cell response is synchronized with the frequency of stimulation with the photoconductive stimulation technique, similar to that observed with field stimulation.

### Spatially selective photoconductive stimulation of live neurons

**Figure 5:**
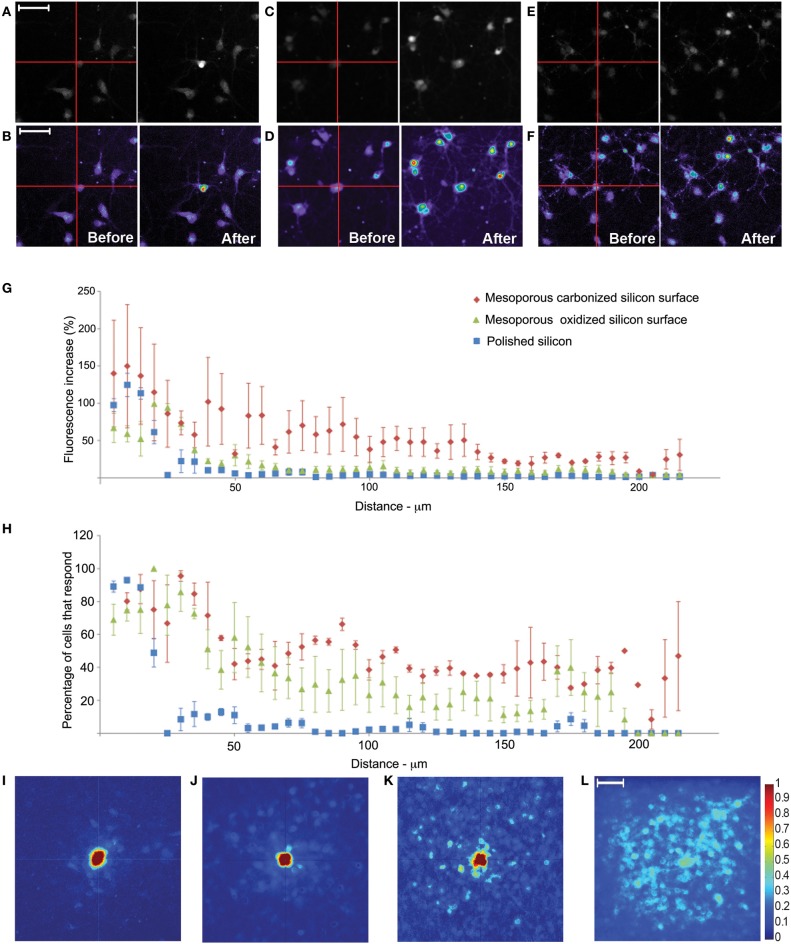
**Spatial resolution of photoconductive stimulation of rat primary cortical neurons on three different substrates**. Representative set of images showing targeted neuronal excitation visualized by Fluo-4 calcium imaging of neurons cultured on **(A,B)** polished silicon, **(C,D)** porous oxidized silicon or **(E,F)** porous carbonized silicon. **(G)** Fluorescence increase as a function of distance to stimulation point and **(H)** percentage of cells activated as a function of distance from stimulation point for neurons cultured on silicon (blue squares), porous oxidized silicon (green triangles) and porous carbonized silicon (red diamonds). Cells were identified in each image using a custom MATLAB script, then binned into 5 μm groups and each graphed point is the average of that group. Error bars show standard error from independent experiments. Cells were counted as activated if fluorescence increased by 15% or more. **(I–L)** Rainbow Heat map representing intensity before and after 5 s of 10 Hz stimulation. Heat map created by averaging fluorescent change in pairs of images with point stimulation at center of frame for neurons cultured on **(I)** polished silicon (*n* = 102 stimulations/3 independent cultures), **(J)** porous oxidized silicon (*n* = 52 stimulations/3 independent cultures) or **(K)** porous carbonized silicon (*n* = 36 stimulations/2 independent cultures). **(L)** Average heat map for neurons cultured on silicon chip with entire field stimulation. Scale bar—50 μm.

One highly desired characteristic of a neuronal stimulation technique is the ability to stimulate neurons in a spatially controlled manner. We measured the ability of the technique to stimulate cells in a spatially confined manner, by manipulating the laser during stimulation. We loaded neurons cultured on the 3 different silicon substrates with Fluo-4, then imaged before and after a 5 s, 10 Hz stimulation train. We used the spot scan function to target the laser at a single cell body and assessed our ability to selectively stimulate that cell. We were usually able to stimulate a single targeted cell on untreated polished silicon, while similar stimulation on the porous oxidized silicon and porous carbonized silicon (Figures 5A–F) resulted in a larger region of stimulation.

Neurons demonstrated robust fluorescence increase in response to stimulation on all three substrates (Figures 5A–F,G). The stimulation on the normal polished silicon substrate resulted in a highly specific region of stimulation with less than 20% of cells that were more than 25 um from the region of stimulation responding. Neurons on the porous oxidized silicon and porous carbonized silicon substrates were less spatially selective with 30% or more cells being activated at 75 um from the point of stimulation. The porous carbonized silicon has the least spatial selectivity of the 3 substrates, with 20% or more of the neurons responding at distances of 200 um from the point of stimulation (Figures 5G,H). We compiled all these images (Figures 5A–F) into heat maps showing average fluorescence change (Figures 5I–K). To assess spatial selectivity on the three silicon based substrates^15^, we applied the same procedure but frame scanned a 320 by 320 μm field. By altering the area scanned by the laser, a larger region of the polished silicon chip can be activated (Figure 5L). Thus, the spatial selectivity of the stimulated region can be altered by use of the desired silicon based substrate or manipulating the area scanned by the laser.

## Discussion

We have designed and implemented a chamber for photoconductive stimulation using a regular inverted confocal microscope. We have demonstrated the applicability of a non-invasive photoconductive stimulation for primary neurons in culture using a laser based photostimulation. More importantly, we have demonstrated that this technique can be spatially manipulated easily by the choice of silicon substrate or altering the area illuminated by the laser. The chamber and technique can be easily implemented on any standard inverted microscope. The system is conducive to both acute and long term studies that that couple neuronal photoconductive stimulation with time-lapse imaging.

The same chamber, technique, and principles can be applied to any excitable cell type, including other types of neurons, neuron-astroctye co-cultures or cardiomyocytes. By combining this technique with other existing technologies, the system can perform cell biological studies that address neuronal network dynamics and dynamic cell biological studies that assess cellular alterations in response to synaptic activity by molecular fluorescent indicators or translocation of neuronal proteins in response to neuronal stimuli.

Our data indicate that silicon based substrates are highly permissive for neuronal growth and photoconductive stimulation. Scaling up of the technique and adapting to multiwall formats would be important advances for drug discovery and characterization based screening techniques. Similarly, by patterning or interspersing the silicon based substrates on glass surfaces, further spatial control over stimulation can be achieved. Thus, this technique is a simple but powerful, versatile and easy to use tool that can be easily implemented in a standard live chamber on an inverted scope with little specialized equipment and is likely to find broad application in cellular and molecular neuroscience.

## Author contributions

Jacob Campbell and Jyothi Arikkath designed experiments, Jacob Campbell performed all photoconductive experiments, Dipika Singh and Jyothi Arikkath devised the technique for freezing primary neurons, Dipika Singh and Jyothi Arikkath characterized the frozen primary neurons, Jyothi Arikkath performed confocal imaging analysis for immunostaining and transfected neurons, Geoffrey Hollett participated in generating silicon substrates under direct supervision of Michael J. Sailor, Shashank M. Dravid performed the electrophysiology experiments. Jyothi Arikkath and Jacob Campbell drafted the manuscript with input from the other authors. Jyothi Arikkath supervised the whole project. This proposal was partly funded by startup funds from the Munroe-Meyer Institute, University of Nebraska Medical Center, an NSF EPSCoR Nebraska Award (EPS-1004094) and an Institutional Development Award (IDeA) from the National Institute of General Medical Sciences of the National Institutes of Health under grant number P20GM12345. Jacob Campbell was partly funded by the UNMC MD/PhD Summer undergraduate research program. All animal experiments were conducted in compliance with approved UNMC protocols.

### Conflict of interest statement

Dr. Arikkath and Dipika Singh are the creators of the freezing media for the primary neurons used in this study and receive royalties from sales of the freezing media. All other authors declare that the research was conducted in the absence of any commercial or financial relationships that could be construed as a potential conflict of interest.
